# Hypoxia-Inducible Factor-1α (HIF-1α) Inhibition Impairs Retinoic Acid-Induced Differentiation in SH-SY5Y Neuroblastoma Cells, Leading to Reduced Neurite Length and Diminished Gene Expression Related to Cell Differentiation

**DOI:** 10.1007/s11064-021-03454-3

**Published:** 2021-09-23

**Authors:** Pedro Ozorio Brum, Guilherme Danielski Viola, Carolina Saibro-Girardi, Camila Tiefensee-Ribeiro, Matheus Ozorio Brum, Juciano Gasparotto, Rachel Krolow, José Cláudio Fonseca Moreira, Daniel Pens Gelain

**Affiliations:** 1 Departamento de Bioquímica, Centro de Estudos em Estresse Oxidativo, Instituto de Ciências Básicas da Saúde, Universidade Federal do Rio Grande do Sul, Porto Alegre, RS Brazil; 2 Laboratório de Programação Neurobiológica do Comportamento Alimentar, Departamento de Bioquímica, Instituto de Ciências Básicas da Saúde, Universidade Federal do Rio Grande do Sul, Porto Alegre, RS Brazil; 3 Laboratório de Medicina Genômica, Centro de Pesquisa Experimental, Hospital de Clínicas de Porto Alegre, Porto Alegre, RS Brazil; 4 Instituto de Ciências Biomédicas, Universidade Federal de Alfenas, Alfenas, MG Brazil; 5 Present Address: Max F. Perutz Labs, University of Vienna, Dr Bohr-Gasse 9, Room 4.510, 1030 Vienna, Austria; 6 New York City, NY USA

**Keywords:** Hypoxia, Differentiation, Neuroblastoma, Cancer, HIF-1, SH-SY5Y

## Abstract

Neuroblastoma is the most common extracranial solid tumour in childhood, originated from cells of the neural crest during the development of the Sympathetic Nervous System. Retinoids are vitamin-A derived differentiating agents utilised to avoid disease resurgence in high-risk neuroblastoma treatment. Several studies indicate that hypoxia—a common feature of the tumoural environment—is a key player in cell differentiation and proliferation. Hypoxia leads to the accumulation of the hypoxia-inducible factor-1α (HIF-1α). This work aims to investigate the effects of the selective inhibition of HIF-1α on the differentiation induced by retinoic acid in human neuroblastoma cells from the SH-SY5Y lineage to clarify its role in cell differentiation. Our results indicate that HIF-1α inhibition impairs RA-induced differentiation by reducing neuron-like phenotype and diminished immunolabeling and expression of differentiation markers.

**Graphic Abstract:**

HIF1A is involved in Retinoic Acid (RA) induced differentiation in SH-SY5Y neuroblastoma cells. siRNA HIF1A gene silencing leads to a weaker response to RA, demonstrated by changes in the neuro-like phenotype and diminished expression of differentiation markers.
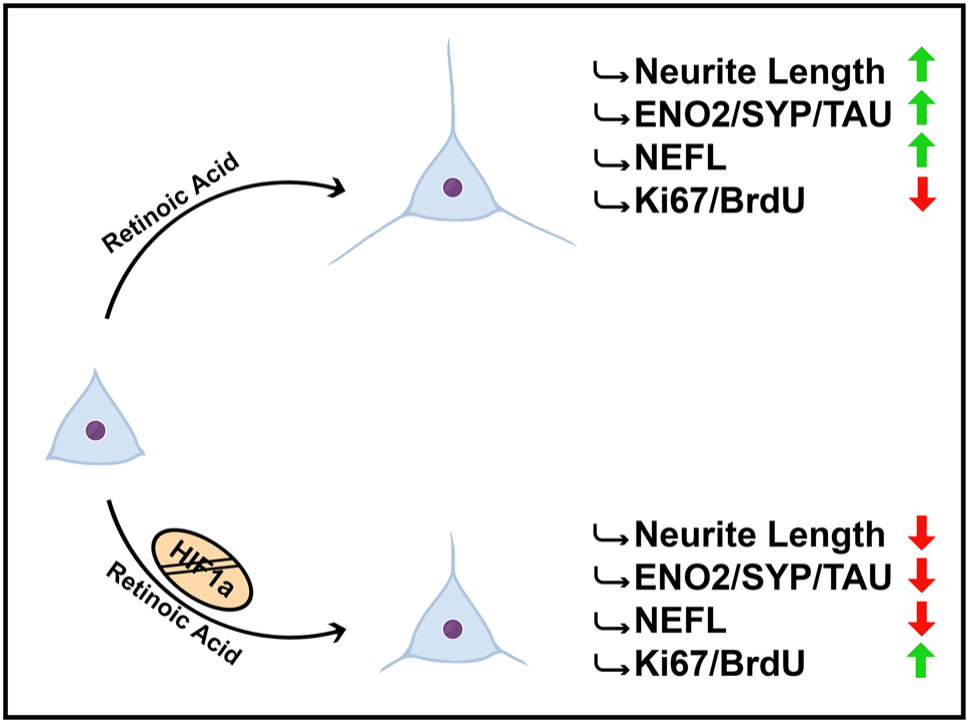

## Introduction

Neuroblastoma (NB) is a heterogeneous paediatric solid tumour with various forms, which range from highly aggressive metastatic cancer—with cure rates as low as < 50%—to low-risk disease—with chances of a good prognostic greater than 90% [1]. NB arises from the Neural crest (NC) during the formation of the Sympathetic nervous system (SNS) [2]. Despite its low incidence, NB is responsible for 10% of childhood cancer deaths [3] and is the most common extracranial solid tumour in childhood [1]. High-risk NB treatment includes high dose myeloablative chemotherapy followed by stem cell rescue, radiation, surgery, and retinoid treatment to avoid cancer resurgence [4, 5].

Hypoxia and hypoxia-inducible factors (HIFs) elevated expression are common features of the tumoural environment [6, 7]. The hypoxia-inducible factor-1 (HIF-1) is a cell-sensor to oxygen concentrations, targeting gene expression related to cell fate. HIF-1 is a heterodimer composed of its α and β subunits. The α subunit’s stability, cellular location, and expression are directly related to oxygen levels. In contrast, the β subunit, also known as Aryl hydrocarbon receptor nuclear translocator (ARNT), is constitutively expressed and is not oxygen-sensible [8]. Once the β subunit dimerises with HIF-1α, HIF-1 binds to Hypoxia-responsive elements (HRE) and promotes gene expression related to angiogenesis, erythropoiesis, metabolism, and cell proliferation and survival. In normoxic conditions, the α subunit is degraded in the von-Hippel-Lindau protein (pVHL) mediated ubiquitin–proteasome pathway, which is inhibited by hypoxia [9]. The interaction between HIF-1α and pVHL is triggered by the post-translational hydroxylation of HIF-1α by Prolyl hydroxylase (PHD) [10]. HIF-PHD hydroxylases HIF-1α in Oxygen-dependent degradation domains (ODD). These post-translational modifications to HIF-1α allow for pVHL binding—an E3 ubiquitin ligase—leading to ubiquitination and further proteasomal degradation [11].

Besides its fine oxygen-dependent regulation, few other factors seem to stabilise HIF-1α. Proinflammatory stimuli may lead to a PI3K/Akt/mTOR-mediated increase in *HIF1A* expression [12]. Moreover, hypoxia-mimetic agents such as deferoxamine (DFO) [13] and CoCl_2_ [14] are capable of stabilising HIF-1α through the inhibition of PHD. Additionally, HIF-1α activity is sensitive to changes within cell energetic metabolism. Citric acid cycle dysfunction and accumulation of its intermediaries leads to inhibition of HIF-1α hydroxylases and therefore results in HIF-1α stabilisation bypassing the oxygen-dependent regulation [15–17]. Interestingly, studies have shown that retinoic acid administration in different cell types leads to HIF-1α accumulation [14, 18] and that RA signalling might depend on HIF-1α [14, 18, 19]. RA treatment in SH-SY5Y leads to changes in cell energetic metabolism, leading to increased oxidative stress [20–22]. Furthermore, elevated levels of oxygen-reactive species activate mitochondrial HIF-1α [23], which may explain the rise in HIF-1α levels after retinoic acid treatment.

Neuroblastoma cells can be induced to differentiate in a neuron-like phenotype. SH-SY5Y neuroblastoma cells express proliferative markers such as the immature neuronal marker nestin and the Proliferating cell nuclear antigen (PCNA). When treated with Retinoic acid (RA), these cells exhibit a neuron-like phenotype with extensive projections, cell-cycle arrest, and the expression of differentiation markers such as enolase 2 (*ENO2*), synaptophysin (*SYP*) and the microtubule-associated protein tau (*MAPT*/*TAU*) [24]. In addition, SH-SY5Y is known for a catecholaminergic phenotype upon differentiation, expressing dopaminergic markers such as tyrosine hydroxylase (*TH*), dopamine transporter (*DAT*) and moderate levels of dopamine-β-hydroxylase (*DßH*) activity [10, 25]. SH-SY5Y neuroblastoma cells exposed to the hypoxia mimetic agent deferoxamine (DFO) exhibit a neuron-like tyrosine hydroxylase (TH) expressing phenotype [13]. In contrast, NB1691 neuroblastoma cells exposed to intermittent hypoxia expressed increased NC markers and decreased SNS markers, even though there was an increase in TH. Additionally, under RA treatment and hypoxia exposure, NB1691 cells demonstrated diminished responsiveness to differentiation induced by RA [26]. In the same study, the authors demonstrated that HIF-1α inhibition under intermittent hypoxia conditions increases a neuron-like phenotype in NB1691 cells. These findings contrast to the work of Cimmino et al. [19], which demonstrated a diminished neuron-like phenotype in HIF-1α silenced neuroblastoma cells - SH-SY5Y, SKNBE2c, SKANS - exposed to RA under normoxic conditions in high serum concentrations (10% FBS).

The purpose of our study was to determine HIF-1α’s role in retinoic acid-induced differentiation in SH-SY5Y cell cultures under normoxic conditions and low serum concentration (1% FBS)—reduced concentrations of FBS inhibit S phase entry, leading cells to remain in G_0_, thus facilitating cell differentiation. For such, we *silenced* HIF-1α expression through small interference RNA (siRNA) and exposed the cell cultures to a 7-day RA-induced differentiation protocol. Our results indicate that HIF-1α inhibition impairs RA-induced differentiation by reducing neuron-like phenotype and diminished immunoreactivity and expression of differentiation markers.

## Methods

### Cell Culture and Differentiation

Human neuroblastoma cells of the lineage SH-SY5Y (passage 21) were obtained from the European Collection of Cell Culture (ECACC) and were grown in DMEM/F12 (Sigma-Aldrich - D8900) containing 10% fetal bovine serum (FBS) at 37 °C in 5% CO_2_ and 95% air in a humidified atmosphere. Differentiation cells were plated at 10^3^ cells/cm^2^ confluency and treated with retinoic acid at 10 µM concentration in DMEM/F12 1% FBS for 7 days with retinoic acid pulses on days 1, 4 and 7.

### siRNA Knockdown

HIF-1α small interference RNA Silencer **®** Select at 5 nM (Ambion - 4392420, ID: n336610) and siRNA scramble (Ambion - AM4635) as control at 30 nM (siRNA concentrations differ as the efficiency of RNA Silencer**®** Select is higher) were transfected using the reverse transfection protocol with siPORT™ NeoFX™ Transfection Agent (Ambion®, Applied Biosystems Inc.) and Opti-MEM following manufacturer’s instructions. Cells were transfected in DMEM/F12 medium with 10% FBS without antibiotics. Knockdown efficiency was evaluated through RT-qPCR and Western Blot. Cells were transfected 24 h before retinoic acid treatment (day 0).

### Treatment Timeline

**Fig. 1:**
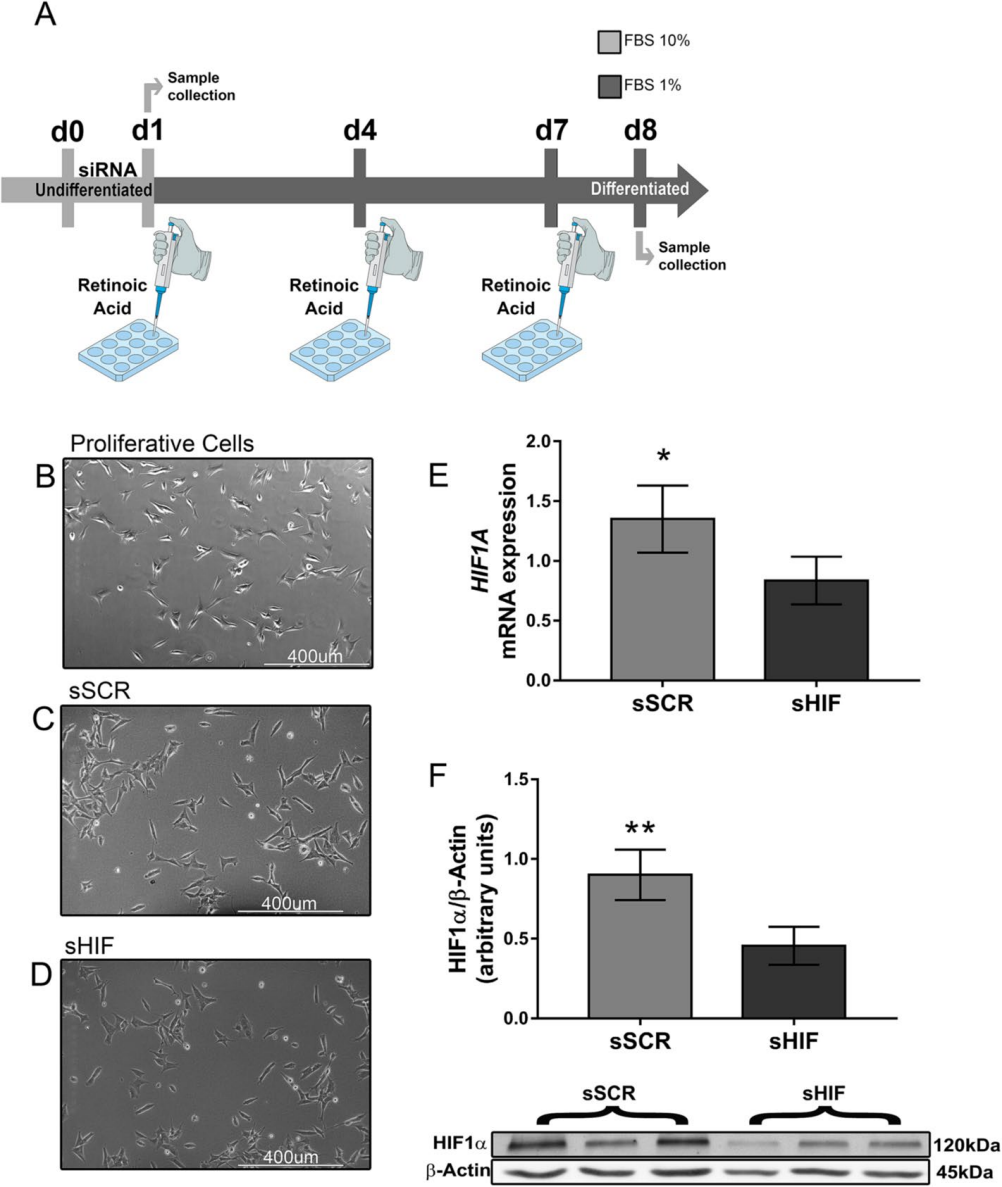
Cell silencing and differentiation protocol **A** Protocols were performed on a 9-day time span, from day 0 to day 7. On day 0, cells were trypsinised and seeded for reverse transfection. On day 1, either cells were collected for RT-qPCR and Western Blot or RA treatment was initiated on the concentration of 10 µM. On days 4 and 7, cells received RA pulses. On day 8, cells were either fixed for immunofluorescence microscopy or collected for RT-qPCR. HIF-1α siRNA transfection leads to a decrease in protein and mRNA levels without morphological alterations that could indicate changes in cell viability. **B** Phase microscopy shows cell morphology in growth conditions before plating and siRNA transfection (d0). Phase microscopy showing cell morphology after siRNA transfection (d1) of both HIF-1α siRNA (sHIF) (**C**) and scramble siRNA (sSCR) (**D**). In **E** HIF-1α mRNA levels (n = 4) and **F** HIF-1α protein levels (n = 6) after transfection. Bars represent mean ± SD (unpaired Student’s t-test *p < 0.01, **p < 0.005)

Experiments were performed on a 9-day timespan beginning on day 0 through day 9 (Fig. 1A). On day 0, cells were trypsinised and transfected with siRNA (siRNA scramble or siRNA HIF1α) at a confluence of 10^3^ cells/cm^2^. After 24 h (day 1), (i) transfection cells were either collected for RT-qPCR or western blot to validate transfection efficiency or (ii) RA treatment was initiated. Retinoic acid pulses were administered within 72 h intervals (days 1, 4 and 7). Cells were either collected for RT-qPCR or fixed for immunofluorescence microscopy 24 h after the last pulse (day 8).

### Western Blot

For western blot analysis, cells were lysed in 4X Laemmli buffer (250 mM Tris, 8% SDS, 40% glycerol and 0.002% bromophenol blue, pH 6.7) then vigorously vortexed and boiled for ten minutes at 100 °C. Samples were loaded and separated in 10% polyacrylamide gel and then electroblotted to nitrocellulose membranes. Protein loading and electroblotting efficiency were verified through Ponceau S staining. Membranes were washed in Tris-buffered saline Tween-20 (Tris 100 mM, pH 7.5, 0.9% NaCl and 0.1% Tween-20) and blocked in TBS-T with 5% BSA. Membranes were washed three times post-blocked and then incubated overnight at 4 °C with primary antibodies (all primary antibodies were used at 1:1000 dilution) for HIF-1α (Cell Signaling - 141795) and β-actin (Sigma-Aldrich - A1978). Subsequently, membranes were incubated with the corresponding species-specific secondary antibody (all secondary antibodies were used at 1:2000 dilution) coupled to peroxidase (Sigma-Aldrich - AP132P; AP124P) following chemiluminescence detection utilising Westar Nova’s 2.0 kit (Cyanagen - XLS071,0250) and GE®’s ImageQuant LAS 4000 CCD camera to obtain images. Western blot analysis was conducted in two experimental sets with a sample size of three biological replicates per group (n = 6). Band densitometry analysis was performed with ImageJ software.

### Immunofluorescence

Cells were fixed in 4% paraformaldehyde for 10 min at room temperature, followed by 10-min permeabilisation utilising ice-cold 0.1% Triton-PBS. To block nonspecific binding, cells were incubated with 1% albumin, 22.52 mg/mL glycine in T-PBS (PBS + Tween 20 0.1%) for 1 h at room temperature. Cells were then incubated overnight with primary antibodies for Glial fibrillary protein (GFAP) (Sigma-Aldrich - G6171), Neurofilament-L (NEFL) (Cell Signalling - 2837), Ki67 (Invitrogen - PA5-19462) and BrdU (Invitrogen - B35128) (all primary antibodies were used at 1:500 dilution), followed by a 1-h incubation with its species-specific corresponding secondary antibody coupled with Alexa Fluor**®** staining (488 nm or 555 nm) from Cell Signalling Technology**®** at room temperature (all secondary antibodies were used at 1:500 dilution). Cells were then incubated for 5 min with DAPI for nucleic acid staining (1:1000; D9542 - Sigma-Aldrich**®**). Following each step, cells were washed three times for 5 min each time in ice-cold PBS. Images were obtained through a Microscopy EVOS® FL Auto Imaging System (AMAFD1000 - Thermo Fisher Scientific**®**). Immunolabeling was measured as the Corrected Total Cell Fluorescence (CTCF) – Queensland Brain Institute at The University of Queensland. We used ImageJ to select cells of interest and calculate cell area and integrated density. CTCF was defined as the difference between Integrated Density (ID) and cell area (A) times mean background fluorescence (B) – CTCF = ID – (A*B). The pictures used for analysis were each taken at the same light exposition with a 40× objective (400× magnification). Colour information was disregarded for CTCF analysis, and Grayscale images were used. Around 50 cells per group were analysed (Undiff. = 51, sSCR + RA = 46, sHIF + RA = 48). Overlapping cells were not considered for analysis to avoid interference.

#### Neurite Total Count and Length

The total number of neurites per cell and the average length of neurites were assessed on NEFL stained cells (40× objective, 400× magnification) using the ImageJ tool, NeuronJ. The total number of neurites per cell was calculated as the (a) sum of all elongated neurite-like NEFL positive stained structures in each replicate divided by (b) the total number of cells. The total number of cells was in turn assessed as the number of DAPI positive structures in the image. The average length of neurites was calculated as (a) the sum of the length (px) of the neurite-like structures divided by (b) the total number of cells in each replicate. Around 150 cells per group were analysed (Undiff. = 143, sSCR + RA = 156, sHIF + 159).

#### BrdU Incorporation Assay

Cells were treated with 10 μM BrdU (Sigma-Aldrich - B5002) in cell medium overnight. Afterwards, cells were fixed with 4% PFA and permeabilised using ice-cold 0.1% Triton-PBS. Later, cells were submitted to DNA hydrolysis with a 10 min treatment using 1 N HCl at 4 °C followed by 10 min 2 N HCl at room temperature. Labelling was conducted using BrdU primary antibody (Invitrogen - B35128). In addition, cells were co-stained with Ki67 (Invitrogen - PA5-19462) for proliferation analysis. Pictures were taken with a 20× objective (200× magnification), and cells were BrdU (Alexa Fluor® 555, red) co-stained with DAPI (blue) were considered as BrdU + . In addition, cells that were Ki67 (Alexa Fluor® 488, green) co-stained with DAPI were considered Ki67 + cells. Experiments were conducted in three biological replicates, and around 500 cells were quantified for each group (Undiff. = 506, sSCR + RA = 488, sHIF + RA = 510).

### Real Time-qPCR

Cells were collected utilising the TRIzol reagent (Thermo Fisher Scientific), and RNA extractions were conducted following the manufacturer's instructions. cDNA synthesis was performed using the High-Capacity cDNA Reverse Transcription kit from Applied Biosystems. Real-time polymerase chain reaction was carried employing SYBR™ Green PCR Master Mix kit, 100 ng of cDNA and 100 µM of each primer: *ENO2*, *SYP*, *TAU*, *HIF1A* and the reference genes *GNB2L* and B2M (Table 1) – Primers were designed across exon-exon borders. Results were normalised in relation to the reference genes (ΔCt), and for each case, the most stable reference gene was applied. Differentiation treatment altered B2M levels slightly; therefore, *GNB2L* was used for normalisation after RA treatment. For each group, four biological replicates were analysed, with three technical replicates for each experiment. Results were expressed using the 2^−ΔΔCT^ method.

**Table 1:**
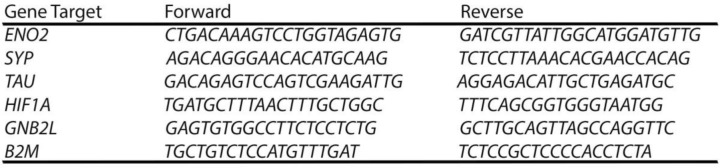
Sequences of the primers employed in RT-qPCR experiments

### Cohort Selection and Gene Expression Analysis

The TARGET RNA-seq dataset and clinical data from Neuroblastoma patients were downloaded from the UCSC Xena browser (https://xenabrowser.net/). High-risk neuroblastoma was defined according to the International Neuroblastoma Risk Group classification as children diagnosed with 18 months old or older and Stage IV disease. Patients with earlier diagnosis and disease stages were included in the Low-risk group. MYCN was not evaluated. The gene expression values were added and then log2-transformed before analysis. The most relevant genes for this study were selected according to their respective Ensembl ID. Only samples clinically classified as stage IV and diagnosis after 18 months old or older were selected for further correlation analysis. Pearson's correlation coefficient was assessed between differentiation-related genes (*NEFL, SYP, MAPT, ENO2*) and *HIF1A*.

### Statistical Analysis

Statistical analysis was performed with the GraphPad Prism software version 7.0 (GraphPad Software Inc., San Diego, USA). When suitable, data was evaluated by one-way ANOVA followed by Tukey’s Multiple Comparison *post-hoc* test or unpaired Student’s t-test. Differences were considered significant when p < 0.05. For RNA-seq data analysis, gene expression was log2-transformed, and Pearson’s correlation coefficient was calculated.

## Results

### siRNA Transfection Effectively Inhibits HIF1α Expression and Diminishes Its Immunocontent

After 24 h from transfection (day 1), cells were collected for Western Blot or RT-qPCR to assess transfection efficiency. mRNA levels (Fig. 1E) and HIF-1α immunocontent (Fig. 1F) decreased when cells were transfected with 5 nM HIF-1α siRNA select (sHIF) in comparison to 30 nM scramble siRNA transfected cells (sSCR) without any morphological alterations (Fig. 1B–D) that could indicate changes in cell viability. In addition, the incubation time used for all experiments was 24 h since it showed greater effectiveness of inhibition when compared to other incubation times (data not shown).

### HIF-1α Silencing Impairs Cell Differentiation Leading to Changes in Cell Morphology

**Fig. 2:**
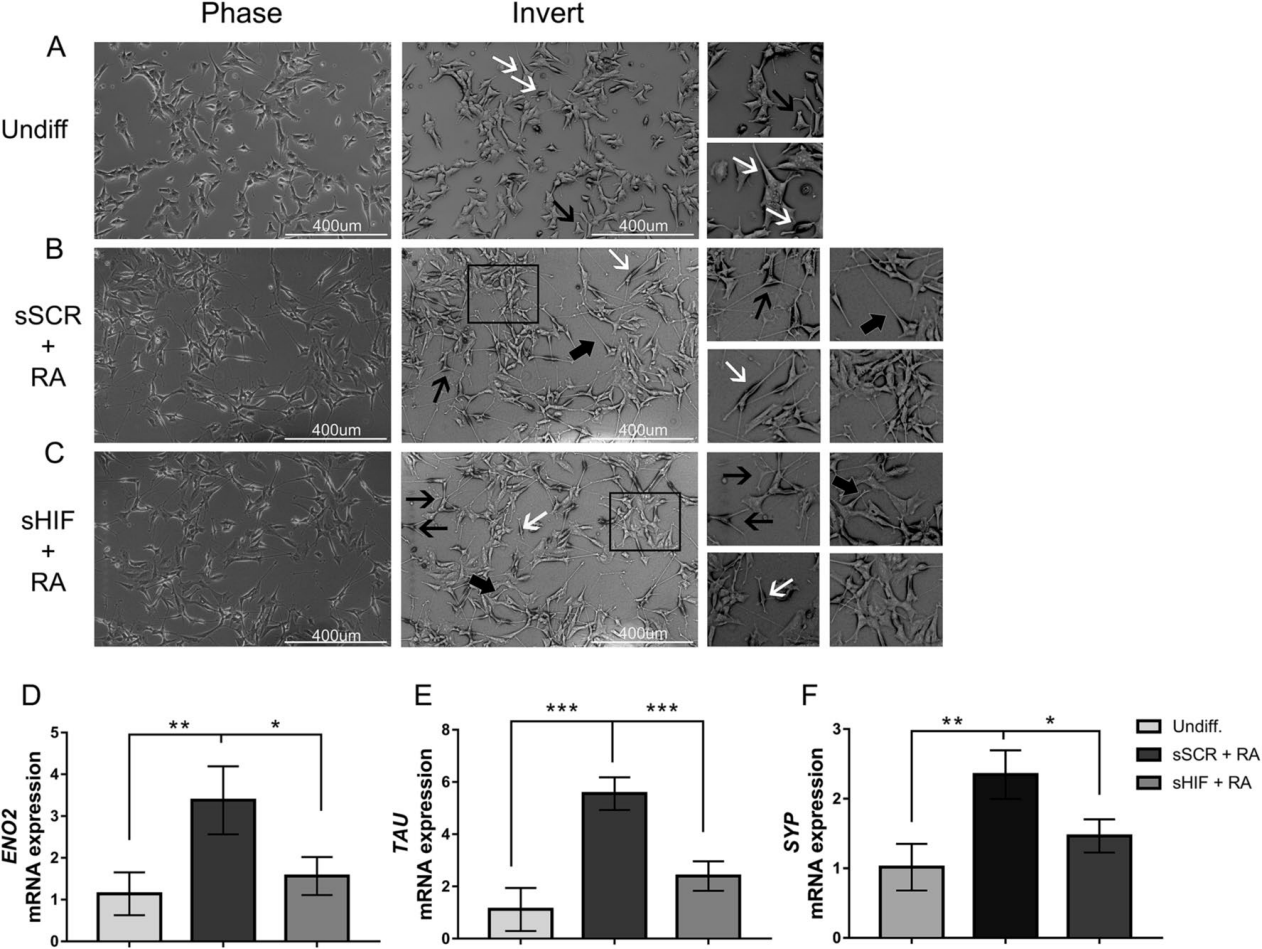
HIF-1α inhibition affects cell differentiation. Black arrows indicate triangular-shaped cells; white arrows indicate flat bipolar cells; thick black arrows indicate elongated unipolar cells; rectangles mark groups of proliferative cells. **A** Phase microscopy of undifferentiated cells demonstrating a triangular-shaped phenotype. **B** sSCR + RA demonstrate extensive projections. **C** sHIF + RA demonstrate shorter projections. Cells were collected for RNA extraction and further cDNA synthesis to analyse neuronal markers expression through RT-qPCR (n = 4). RA treatment leads to an increase in **D** *ENO2*, **E** *SYP*, **F** *TAU* in sSCR + RA compared to undifferentiated cells and sHIF + RA cells. HIF-1α *silencing* was able to reduce differentiation-related gene expression. Bars represent mean ± SD (one-way ANOVA followed by Tukey’s Multiple Comparison *post-hoc* test *p < 0.05, **p < 0.01 ***p < 0.001)

**Fig. 3:**
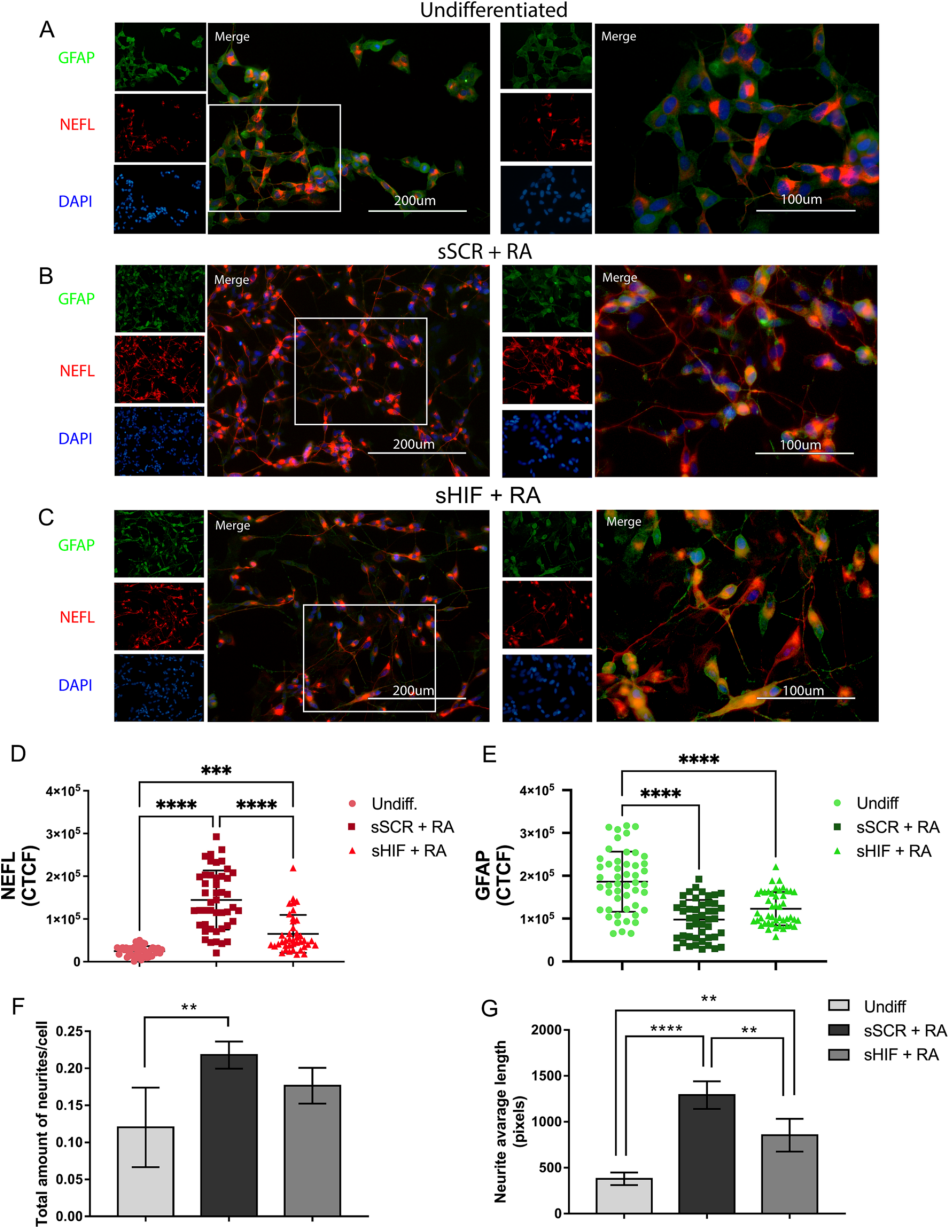
HIF-1α inhibition led to diminished levels of NEFL and a lower average neurite length. Cells were stained for immunofluorescence with anti-NEFL antibody (red) as a neuronal marker and anti-GFAP antibody (green) as a glial marker. **A** shows the undifferentiated (undiff.) cell morphology, **B** shows sSCR + RA cells, **C** correspond to sHIF + RA cells. **D** and **E** show the fluorescence intensity of NEFL and GFAP, respectively. sSCR + RA cells demonstrated greater levels of NEFL immunoreactivity in comparison to Undifferentiated and sHIF + RA cells. Undiff. cells displayed higher GFAP immunoreactivity than sSCR + RA and sHIF + RA. Each symbol represents the Corrected total cell fluorescence (CTCF) of a single cell. Error bars represent mean ± SD. **F** and **G** show the total amount of neurites/cells and the average neurite length, respectively. Undiff. cells demonstrated a lower amount of neurites than sSCR + RA. Furthermore, sSCR + RA cells displayed a higher neurite average length. Although, sHIF + RA cells had an average neurite length higher than undiff. cells. Columns represent mean ± SD (one-way ANOVA followed by Tukey’s Multiple Comparison post-hoc test *p < 0.05, **p < 0.005, ***p < 0.0003, ****p < o.0001)

Transfected cells were treated with 10 µM retinoic acid for 7 days. RA pulses were administered on days 1, 4 and 7 (Fig. 1A). Undifferentiated (undiff.) cells demonstrated a triangle-shaped morphology (Fig. 2A) with a low total neurites/cell ratio (Fig. 3F) and low average neurite length (Fig. 3G). sSCR cells treated with retinoic acid (sSCR + RA) demonstrated extensive neurites (Fig. 2B) with a greater neurite length average and total neurite/cell (Fig. 3F–G). HIF-1α *silencing* (sHIF + RA) was capable of reducing the neuron-like phenotype induced by retinoic acid (Fig. 2C), demonstrating significantly shorter neurites (Fig. 3G) but was not capable of diminishing the total amount count per cell (Fig. 3F). These results suggest that HIF1-1α *silencing* attenuates neurite elongation rather than neurite outgrowth.

### Downregulation of TAU, ENO2 and SYP in Response to HIF-1α Inhibition

To assess whether HIF-1α inhibition could alter the expression of differentiation markers related to the retinoic acid treatment, we performed RT-qPCR for *TAU, ENO2* and *SYP.* As we observe in Fig. 2D–F, the expression of differentiation markers was reduced in sHIF + RA cells compared to sSCR + RA cells (*TAU*: p = 0.0148; *ENO2*: p = 0.0390; *SYP*: p = 0.0295). However, this change was not as marked as that between the undifferentiated proliferative cells (Undiff.) and sSCR + RA cells (*TAU*: p = 0.0001; *ENO2*: p = 0.0096; *SYP*: p = 0.0049). These results indicate that HIF-1α inhibition reduces the expression of several neuronal markers.

### Inhibition of HIF-1α Leads to Diminished NEFL Immunoreactivity without Changes in GFAP Levels

Cells were prepared for immunofluorescence staining to analyse NEFL immunoreactivity as a neuro-differentiation marker. In addition, cells were co-stained for the glial marker GFAP, as previous findings [5] reported glial trans-differentiation induced by retinoic acid in HIF-1α inhibited neuroblastoma cells. As we can observe in Fig. 3A–C, there were morphological changes between undiff., sSCR + RA and sHIF + RA cells. The undiff. group showed a high proliferative phenotype with a strong GFAP signal, no projections, and a larger cell body area (Fig. 3A). sSCR + RA cells demonstrated a highly differentiated phenotype with extensive projections and strong reactivity to NEFL antibody (Fig. 3B). sHIF + RA showed reduced NEFL immunoreactivity and shorter projections (3C). sSCR + RA cells demonstrated greater NEFL immunoreactivity when compared to both undiff. and sHIF + RA (Fig. 3D). GFAP signal was elevated in undiff. cells, when compared to both sSCR + RA and sHIF + RA (Fig. 3E). These results indicate that HIF-1α inhibition weakens the signal of NEFL immunolabeling. Nonetheless, HIF-1α inhibition was not capable of increasing GFAP labelling as previous reports [19].

### HIF-1α Inhibition Affects the Proliferative Status of RA Treated Cells

**Fig. 4:**
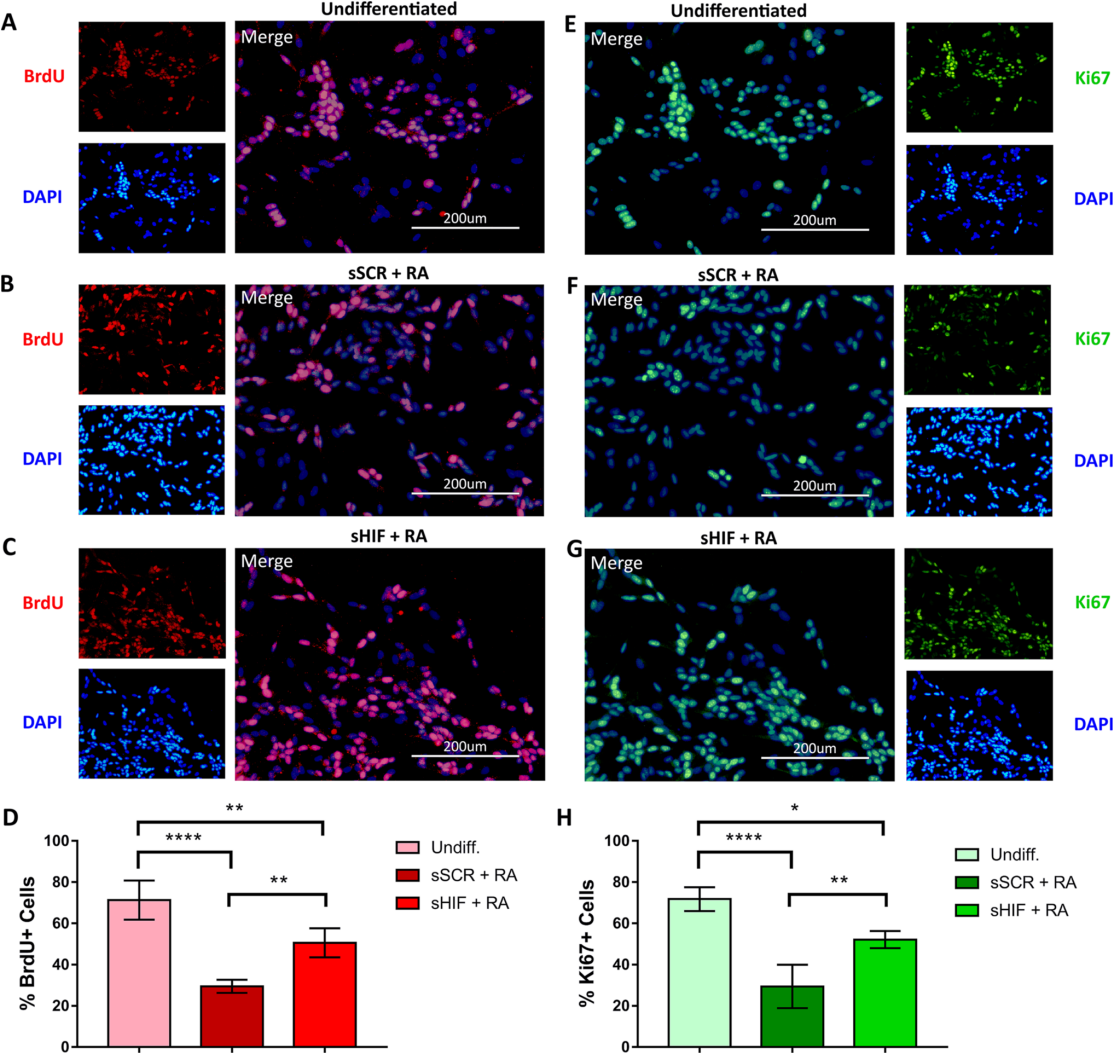
RA-induced reduction of proliferation is less dramatic in sHIF + RA cells. SH-SY5Y cells were co-stained for the proliferative markers BrdU and Ki67. The amount of BrdU + cells in the undiff. **A** The group was higher than both sSCR + RA (**B**) and sHIF + RA (**C**) cells. However, inhibition of HIF-1α in sHIF + RA cells leads to a partial recovery of the proliferative status with an increased amount of BrdU positive cells than sSCR + RA (**D**). Amount of Ki67 + cells in the groups Undiff. (**E**), sSCR + RA (**F**) and sHIF + RA (**G**) followed the same pattern (**H**) as for BrdU +

SH-SY5Y cells were co-stained with BrdU and Ki67 to assess the proliferative status. Undiff. cells displayed a higher amount of BrdU + cells (n = 5, 71,3% ± 9,5 BrdU +). sSCR + RA group had a less proliferative status (29,5% ± 3,9 BrdU +) whilst sHIF + RA cells partially rescued the proliferative status of Undiff. cells (50,6% ± 8,6 BrdU +) (Fig. 4A–D). A similar pattern was observed for Ki67 staining, as undiff. cells displayed a higher amount of Ki67 + cells (72,9% ± 5,8), whereas sSCR + RA had a dramatic reduction in Ki67 + cells (29,4% ± 10,5). sHIF + RA partially recovered the Ki67 + pattern of undiff. cells (52,1% ± 5,1) (Fig. 4F–I).

### NEFL and SYP Expression is Related to HIF1A Expression in Neuroblastoma Patients

**Fig. 5:**
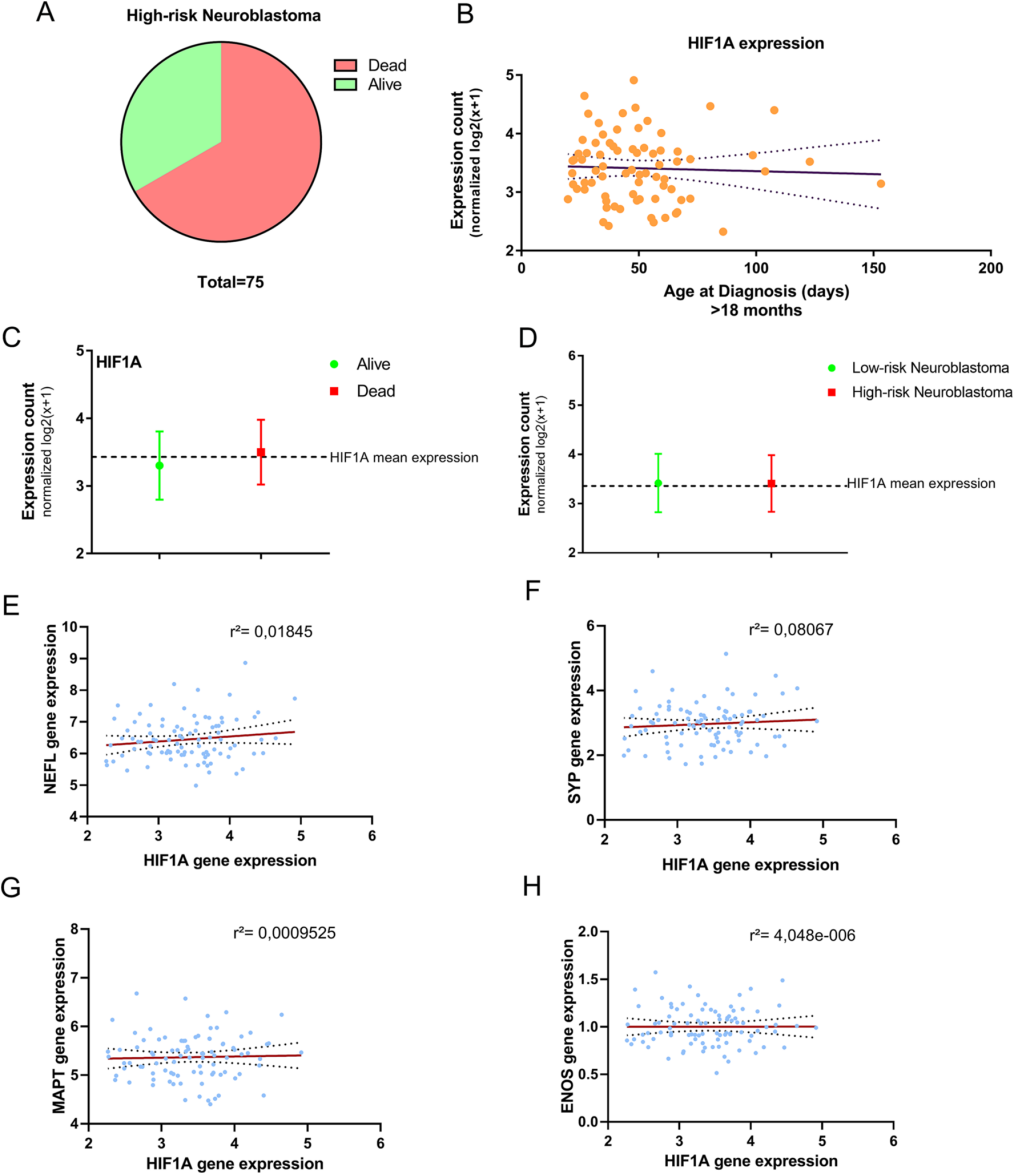
*HIF1A* expression was analysed in a cohort of 75 High-Risk (**A**) and 10 Low-Risk Neuroblastoma patients. Age of diagnosis (from 18 months) did not correlate with *HIF1A* expression (**B**). *HIF1A* mean expression remained unaltered among deceased and alive patients (**C**) and low-risk and high-risk patients. We evaluated the normalised gene expression of differentiation markers *NEFL* (**E**), *SYP* (**F**), *MAPT* (**G**) and *ENO2* (**H**) and correlated to HIF1A normalised gene expression. There was no correlation between differentiation-related gene expression and *HIF1A* expression

Hypoxia and the overexpression of hypoxia-inducible factors are common features of aggressive tumours. To analyse the relation between *HIF1A* expression and differentiation-related genes in high-risk neuroblastoma patients, we looked into gene expression data from the TARGET database. Patients were first classified as High-risk or low-risk neuroblastoma (see Cohort Selection and Gene Expression Analysis Methods). Then, both deceased and alive patients were taken into consideration for analysis (Fig. 5A). According to the International Neuroblastoma Risk Group classification, time of diagnosis is a crucial feature for categorising high-risk neuroblastoma. Therefore, we analysed whether later neuroblastoma diagnosis correlated with increased HIF1A expression. No correlation was observed (Fig. 5B). Furthermore, HIF1A mean expression was not altered either among deceased and alive patients (Fig. 5C) or High-risk and Low-risk neuroblastoma patients (Fig. 5D).

We further analysed the expression of differentiation-related genes (*SYP*, *NEFL*, *ENO2*, *MAPT*) in high-risk neuroblastoma patients and correlated them to HIF1A expression. However, no correlation was found for the four differentiation-related genes analysed (Fig. 5E–H).

## Discussion

HIF-1α role in neuroblastoma cells differentiation treatment is ambiguous. Whereas in hypoxic conditions, HIF-1α may be responsible for RA resistance [26], it might be required for differentiation towards a neuron-like phenotype under normoxic conditions, as seen in previous findings by Cimmino et al. [19]. In this study, we evaluated the role of HIF-1α in RA-induced differentiation in neuroblastoma cells of the lineage SH-SY5Y under normoxic conditions. Our results demonstrate that HIF-1α inhibition could impair the differentiation properties of RA, leading to a reduced expression of differentiation markers and altered morphological features.

By RT-qPCR, we demonstrated that differentiation markers such as *SYP*, *TAU* and *ENO2* were downregulated in sHIF + RA cells, even though differentiation marker presence was still higher than in undifferentiated cells. Interestingly, *SYP* and *TAU* mean expression was elevated in HIF1A High Expression neuroblastoma patients. Our results complement Cimmino et al*.* [19], who have shown that the inhibition of HIF-1α leads to a reduced expression of differentiation markers *TUJ-1* and *NEFL* in retinoic acid-treated SH-SY5Y cells with lower neurite outgrowth and average neurite length. Even though we could not see a significant difference in total neurite/cell between sSCR + RA and sHIF + RA cells, we can observe an upward tendency in the number of neurites/cells in the sSCR + RA group. Previous studies have linked hypoxia and neuronal differentiation [19, 20]. HIF-1α and PHD regulate the Rho-associated protein kinase (ROCK), as inhibition of ROCK is linked to neurite outgrowth and elongation [27, 28]. HIF-1α activation through CoCl_2_ administration in mesenchymal stem cells leads to inhibition of ROCK concomitant with morphological changes and increased neuronal markers [27]. This relationship between HIF-1α and Rho/ROCK might explain the diminished neurite length on HIF-1α *silenced* cells.

By immunofluorescence microscopy, we were able to show that HIF-1α’s inhibition partially impairs retinoic acid-induced differentiation, showing morphological alterations such as a reduced average length of neurites combined with a lower NEFL signal. However, we could not observe a significant difference in GFAP levels in sHIF + RA cells. Cimmino et al. [19] proposed that HIF-1α inhibition leads to enhanced glial trans-differentiation in SH-SY5Y due to an enhanced GFAP immunocontent in a 25-day treatment in high-serum concentration. In a shorter treatment (7 days) and lower serum concentrations, we did not observe the same alterations in GFAP levels. As previous flow cytometry experiments reported [29], RA treatment decreases neuroblastoma cell proliferation. Investigating cell proliferation using BrdU incorporation and Ki67 proliferation marker, we observed that HIF-1a inhibition affects these changes in the proliferative status induced by RA. Therefore, HIF-1α expression is relevant for the initiation of differentiation, whereas its inhibition enhances proliferation.

The role of RA in cell proliferation and differentiation is linked to HIF-1α with different responses in different cell types and conditions. Zhang et al*.* [14] have demonstrated an increase in HIF-1α protein in response to retinoic acid treatment in myeloid leukemic cells. HIF-1α conditional induction significantly increased cell differentiation, whereas HIF-1α inhibition with short hairpin RNA significantly decreased cell differentiation. In contrast, in neural stem and progenitor cells of the adult hippocampus, retinoic acid has a role in maintaining cell proliferation through the regulation of S-phase re-entry [18]. Moreover, retinoic acid is known for its differentiating effect on neuroblastoma cells [10, 24, 25, 30]. Oxygen levels may regulate retinoic acid-HIF-1α’s influence in neuroblastoma cells differentiation. Bhaskara et al. [26] have shown that in an intermittent hypoxia (IH) model, retinoic acid reduces the differentiation towards a neuron-like phenotype. Furthermore, the authors demonstrated that HIF-1α inhibition in IH is capable of inducing neuron-like characteristics.

Contrasting to Bhaskara et al. [26], the current study demonstrates that HIF-1α inhibition in normoxic conditions attenuates the retinoic acid differentiation in neuroblastoma cells, reducing average neurite length, NEFL immunoreactivity, and *SYP, TAU* and *ENO2* expression. Cimmino et al. [19] corroborate our results, showing a reduction in cell differentiation towards a neuron-like phenotype. However, Cimmino and collaborators [19] assert that morphological alterations such as the appearance of flat cells forming ganglion-like structures concurrent with an enhanced GFAP signal indicate that HIF-1α inhibition followed by RA treatment leads to enhanced glial trans-differentiation. It is essential to add that SH-SY5Y cell clones display different phenotypes depending on passage number, cell culture medium, serum concentration, and handling. Cimmino et al. [19] employed a longer-duration protocol with higher serum concentrations (10% FBS); these methodological singularities might justify GFAP signal elevation and the emergence of ganglion-like structures.

Several authors commonly use markers such as NEFL, TAU, SYP and ENO2 to determine neuroblastoma cell differentiation in vitro [19, 20, 29]. While these markers in vitro may suggest success in retinoic acid treatment, they may have different relevance for neuroblastoma patients. Immunohistochemistry of neuroblastoma tumours demonstrates that (i) synaptophysin is constitutively expressed by neuroblastoma tumoural cells, (ii) *NEFL* gene mutation is common yet inefficient in neuroblastoma tumours, and (iii) neuron-specific enolase (*ENO2*) is a non-favourable marker. Analysing a cohort of 75 high-risk neuroblastoma, we could not observe any correlation between differentiation-related genes and *HIF1A* expression (Fig. 5E–F). Further studies are needed to explain HIF-1α’s role in retinoid therapies and neuroblastoma treatment.

## Conclusion

In conclusion, we show that HIF-1α inhibition partially inhibits retinoic acid-induced differentiation in SH-SY5Y cells. HIF-1α silencing was able to significantly change the morphology of retinoic acid-treated neuroblastoma cells and reduce the expression of differentiation markers. In addition, HIF-1α proved itself vital for the RA-induced reduction of proliferation in SH-SY5Y treated cells. These findings suggest that HIF-1α is involved in retinoic acid-induced differentiation regulation and may provide grounds for further studies about this transcription factor role in cell differentiation.

The tumoural microenvironment presents fluctuations in oxygen levels within the tumour; hence, HIF-1α has a vital role as an oxygen sensor in cancer cells. Understanding how HIF-1α influences cell proliferation and differentiation may provide the basis for better comprehension of its influence in retinoid therapies for the treatment of neuroblastoma.

## Data Availability

Specific datasets used and analysed in the current study are available from the corresponding author on reasonable request.

## References

[1] Whittle SB, Smith V, Doherty E, Sibo Z, McCarty S, Zage PE (2017). Overview and recent advances in the treatment of neuroblastoma. Expert Rev Anticancer Ther.

[2] Huertas-Castaño C, Gómez-Muñoz MA, Pardal R, Vega FM (2020). Hypoxia in the initiation and progression of neuroblastoma tumours. Int J Mol Sci.

[3] Smith MA, Seibel NL, Altekruse SF, Ries LAG, Melbert DL, O’Leary M, Smith FO, Reaman GH (2010). Outcomes for children and adolescents with cancer: challenges for the twenty-first century. J Clin Oncol.

[4] Hämmerle B, Yañez Y, Palanca S, Cañete A, Burks DJ, Castel V, Font de Mora J (2013). Targeting neuroblastoma stem cells with retinoic acid and proteasome inhibitor. PLoS ONE.

[5] Coughlan D, Gianferante M, Lynch CF, Stevens JL, Harlan LC (2017). Treatment and survival of childhood neuroblastoma: evidence from a population-based study in the United States. Pediatr Hematol Oncol.

[6] Jing X, Yang F, Shao C, Wei K, Xie M, Shen H, Shu Y (2019). Role of hypoxia in cancer therapy by regulating the tumour microenvironment. Mol Cancer.

[7] Petrova V, Annicchiarico-Petruzzelli M, Melino G, Amelio I (2018). The hypoxic tumour microenvironment. Oncogenesis.

[8] Lee J, Bae S, Jeong J, Kim S, Kim K (2014). Hypoxia-inducible factor (HIF-1)α: its protein stability and biological functions. Exp Mol Med.

[9] Marxsen JH, Stengel P, Doege K, Heikkinen P, Jokilehto T, Wagner T, Jelkmann W, Jaakkola P, Metzen E (2004). Hypoxia-inducible factor-1 (HIF-1) promotes its degradation by induction of HIF-α-prolyl-4-hydroxylases. J Biochem.

[10] Shipley MM, Mangold CA, Szpara ML (2016). Differentiation of the SH-SY5Y human neuroblastoma cell line. J Vis Exp.

[11] Moroz E, Carlin S, Dyomina K, Burke S, Thaler HT, Blasberg R, Serganova I (2009). Real-time imaging of HIF-1a stabilization and degradation. PLoS ONE.

[12] Iommarini L, Porcelli AM, Gasparre G, Kurelac I (2017). Non-canonical mechanisms regulating hypoxia-inducible factor 1 alpha in cancer. Front Oncol.

[13] Lim J, Kim H, Bang Y, Seol W, Choi HS, Choi HJ (2016). Hypoxia-inducible factor-1α upregulates tyrosine hydroxylase and dopamine transporter by nuclear receptor ERRγ in SH-SY5Y cells. NeuroReport.

[14] Zhang J, Song L, Huang Y, Zhao Q, Zhao K, Chen G (2008). Accumulation of hypoxia-inducible factor-1α protein and its role in the differentiation of myeloid leukemic cells induced by all-trans retinoic acid. Haematologica.

[15] Esfahani M, Karimi F, Afshar S, Niknazar S, Sohrabi S, Najafi R (2015). Prolyl hydroxylase inhibitors act as agents to enhance the efficiency of cell therapy. Expert Opin Biol Ther.

[16] Selak MA, Armour SM, MacKenzie ED, Boulahbel H, Watson DG, Mansfield KD, Pan Y, Simon MC, Thompson CB, Gottlieb E (2005). Succinate links TCA cycle dysfunction to oncogenesis by inhibiting HIF-alpha prolyl hydroxylase. Cancer Cell.

[17] Koivunen P, Hirsilä M, Remes AM, Hassinen IE, Kivirikko KI, Myllyharju J (2007). Inhibition of hypoxia-inducible factor (HIF) hydroxylases by citric acid cycle intermediates. J Biol Chem.

[18] Mishra S, Kelly KK, Rumian NL, Siegenthaler JA (2018). Retinoic acid is required for neural stem and progenitor cell proliferation in the adult hippocampus. Stem Cell Rep.

[19] Cimmino F, Pezone L, Avitabile M, Acierno G, Andolfo I, Capasso M, Iolascon A (2015). Inhibition of hypoxia inducible factors combined with all-trans retinoic acid treatment enhances glial transdifferentiation of neuroblastoma cells. Sci Rep.

[20] de Ramos VM, Zanotto-Filho A, de Bittencourt Pasquali MA, Klafke K, Gasparotto J, Dunkley P, Gelain DP, Moreira JCF (2016). NRF2 mediates neuroblastoma proliferation and resistance to retinoic acid cytotoxicity in a model of in vitro neuronal differentiation. Mol Neurobiol.

[21] de Bittencourt Pasquali MA, de Ramos VM, Albanus RD (2016). Gene expression profile of NF-κB, Nrf2, glycolytic, and p53 pathways during the SH-SY5Y neuronal differentiation mediated by retinoic acid. Mol Neurobiol.

[22] Kunzler A, Zeidán-Chuliá F, Gasparotto J (2017). Changes in cell cycle and up-regulation of neuronal markers during SH-SY5Y neurodifferentiation by retinoic acid are mediated by reactive species production and oxidative stress. Mol Neurobiol.

[23] Li HS, Zhou YN, Li L, Li SF, Long D, Chen XL, Zhang JB, Feng L, Li YP (2019). HIF-1α protects against oxidative stress by directly targeting mitochondria. Redox Biol.

[24] Kovalevich J, Langford D (2013). Considerations for the use of SH-SY5Y neuroblastoma cells in neurobiology. Methods Mol Biol.

[25] Filograna R, Civiero L, Ferrari V, Codolo G, Greggio E, Bubacco L, Beltramini M, Bisaglia M (2015). Analysis of the catecholaminergic phenotype in human SH-SY5Y and BE(2)-M17 neuroblastoma cell lines upon differentiation. PLoS ONE.

[26] Bhaskara VK, Mohanam I, Rao JS, Mohanam S (2012). Intermittent hypoxia regulates stem-like characteristics and differentiation of neuroblastoma cells. PLoS ONE.

[27] Pacary E, Tixier E, Coulet F, Roussel S, Petit E, Bernaudin M (2007). Crosstalk between HIF-1 and ROCK pathways in neuronal differentiation of mesenchymal stem cells, neurospheres and in PC12 neurite outgrowth. Mol Cell Neurosci.

[28] Miyake S, Muramatsu R, Hamaguchi M, Yamashita T (2015). Prolyl hydroxylase regulates axonal rewiring and motor recovery after traumatic brain injury. Cell Death Discov.

[29] Girardi CS, Rostirolla DC, Lini FJM, Brum PO, Delgado J, Tiefensee-Ribeiro C, Teixeira AA, Peixoto DO, Heimfarth L, Kunzler A, Moreira JCF, Gelain DP (2019). Nuclear RXRα and RXRβ receptors exert distinct and opposite effects on RA-mediated neuroblastoma differentiation. Biochim et Biophys Acta Mole Cell Res.

[30] Encinas M, Iglesias M, Liu Y, Wang H, Ceña V, Gallego C, Comella JX (2000). Sequential treatment of SH-SY5Y cells with retinoic acid and brain-derived neurotrophic factor gives rise to fully differentiated, neurotrophic factor-dependent, human neuron-like cells. J Neurochem.

